# Mitofusin 2 regulates the oocytes development and quality by modulating meiosis and mitochondrial function

**DOI:** 10.1038/srep30561

**Published:** 2016-07-29

**Authors:** Qun Liu, Lina Kang, Lingjuan Wang, Ling Zhang, Wenpei Xiang

**Affiliations:** 1 Family Planning Research Institute, Tongji Medical College, Huazhong University of Science and Technology, Wuhan 430030, China

## Abstract

Mitofusin-2 (Mfn2), one of the mitochondrial dynamic proteins plays a key role in maintaining the integrity of mitochondrial morphology and function. However, it is unknown if Mfn2 influences the quality of oocytes in the process of development by modulating mitochondrial function *in vitro*. In this study, immature oocytes were transfected with Mfn2-siRNA for 16 h. We found that the expression level of the *Mfn2* gene was significantly lower than those of the control group. The rates of maturation and fertility were also found to have declined. Moreover, mitochondrial structure and function, especially the morphogenesis of spindles, were observed as abnormal during meiosis. Thus, the above findings indicate that down-regulation of Mfn2 may have an impact on the maturation and fertilization of immature oocytes *in vitro* by modulating meiosis and mitochondrial function.

Mitochondria, energy generating organelles, are crucial for most somatic cells^1^. Mitochondria are involved in eukaryotic cell metabolism, calcium homeostasis and signal transduction^2^. Generally, mitochondrial divisions and fusions are modulated by some proteins such as Drp1 and Fis1, which are the key fission mediators, as well as by OPA1, Mfn1 and Mfn2, which are fusion mediators in mammals. More importantly, a mass of vital functions of mitochondria links to their shapes that are fusion or fission^3^,^4^. Many cellular activities, such as stress responses, cell metabolism, and cell death are normal when mitochondria fused, however, cellular dysfunction occurs when mitochondria are fission^5^.

Mitofusion-2 (Mfn2) is a GTPase, which widely distributes on the outer mitochondrial membrane. It not only controls mitochondrial fusion and tethering, but also maintains normal cellular functions in mammals by regulating the electron transport chain of aerobic respiration, mitochondrial membrane potential, cellular metabolism and apoptosis^6^,^7^. Mutations of the *Mfn2* gene can cause the genetic neurodegenerative disease Charcot-Marie-Tooth 2A^8^. Decreasing Mfn2 levels have been proven to be related to vascular proliferation perturbations^9^. Mfn2 has also been indicated to be essential to the development of mice embryos^10^. Additionally, a low level of Mfn2 protein in placental villi cells has been revealed to correlate with spontaneous abortions in human women^11^.

Oocytes contain mitochondria for the germination of immature oocytes, which requires a great deal of energy^12^. The distribution of mitochondria varies within different developmental stages of oocytes^13^, and the protein syntheses and energy production from mitochondria play an important role in meiotic recovery and maturation of GV oocytes^14^. Any shortage of ATP produced by mitochondria during the growth of the oocytes will affect their development^14^. However, whether Mfn2 has any effects on oocyte development and quality and its related mechanisms remains unknown. The main goal of this study is to investigate the effect of Mfn2 on oocyte development *in vitro* and further explore its detailed mechanisms.

## Results

### Mfn2 expression in oocytes after transfection

**Figure 1:**
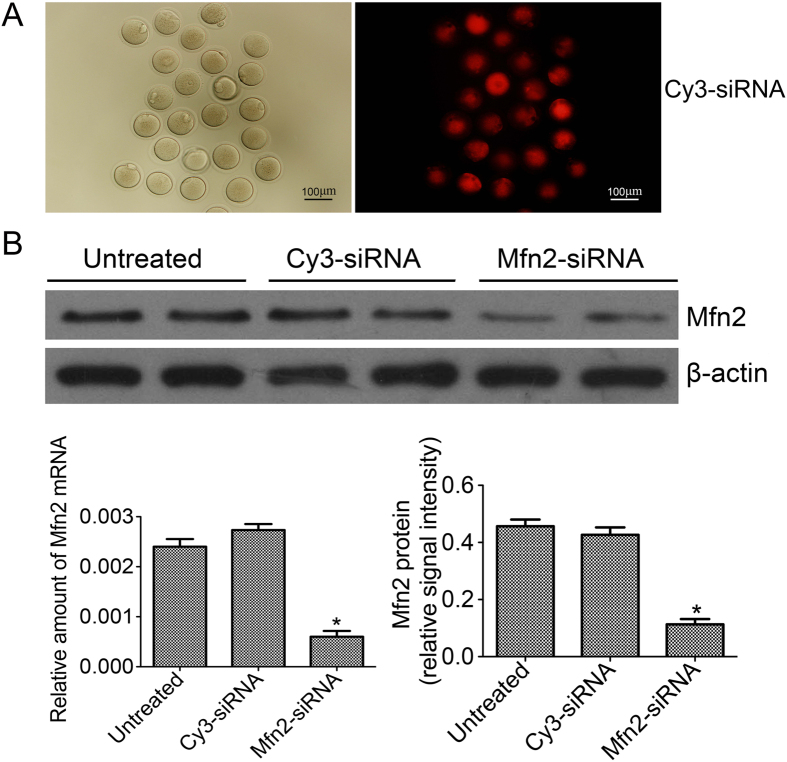
Mfn2 levels of oocytes matured *in vitro* in three groups after transfection. (**A**) The most representative image of transfection efficiency obtained by inverted fluorescence microscope (subcellular distribution of Cy3-siRNA, red); (**B**) The relative amounts of Mfn2 protein and mRNA levels of oocytes in three groups. *P < 0.05 vs. Untreated and Cy3-siRNA groups.

We first investigated the transfection efficiency by using Cy3-siRNA and observed the red fluorescence inside oocytes clearly (Fig. 1A), which proved siRNA had been transfected into the oocytes. After oocytes matured *in vitro* in three groups (Mfn2-siRNA, Cy3-siRNA, untreated groups), we measured the Mfn2 protein and mRNA levels in MII oocytes by western blotting and PCR, respectively. The results show that the levels of both protein and mRNA in the Mfn2-siRNA transfected group were significantly less than that in the Cy3-siRNA and untreated groups (*P* < 0.05) (Fig. 1B).

### Knockdown of *Mfn2* affected oocytes maturation and fertilization *in vitro*

**Figure 2:**
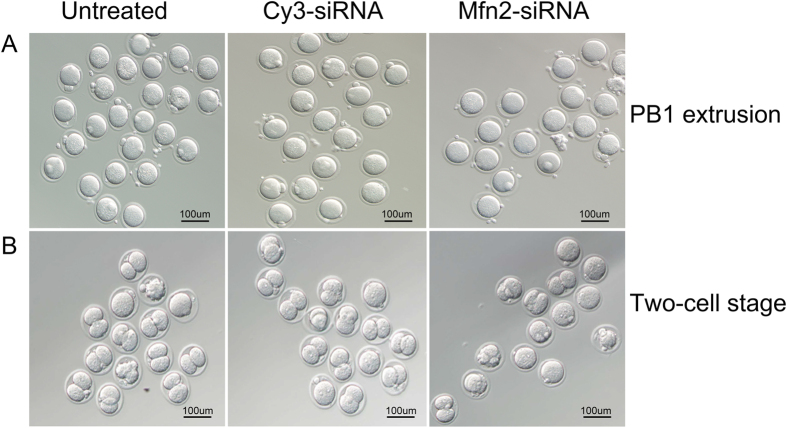
Oocytes maturation and fertilization *in vitro* after transfection. (**A**) The comparative image of PB1 extrusion in untreated, Cy3-siRNA and Mfn2-siRNA groups; (**B**) The representative image of Two-cell stage fertilized eggs in three groups. Date were means ± SEM of three separate experiments.

To address the Mfn2 effect on oocytes, GV stage oocytes were treated with Mfn2-siRNA and Cy3-siRNA *in vitro* and were incubated to the MII stage. After IVM for 16 h, the cumulus cells in the Mfn2-siRNA group expanded drastically when compared with those in the Cy3-siRNA group, and the extrusion of the first polar body (PB1) was obviously lower in the Mfn2-siRNA group (54.7%) than in the Cy3-siRNA group (78.8%) and untreated group (81.3%) (Fig. 2A, Table 1) (P < 0.05). Meanwhile, oocytes matured *in vitro* were fertilized and developed to two-cell stage eggs after 24 h. The results indicated that a knockdown of Mfn2 influenced fertilization and cleavage of oocytes. The fertilization and cleavage rate in Mfn2-siRNA group (61.0%) was dramatically decreased when compared with the Cy3-siRNA group (76%) and the untreated group (77.5%) (Fig. 2B, Table 2) (P < 0.05).

### Low expression of *Mfn2* caused mitochondrial dysfunction in mouse oocytes

**Figure 3:**
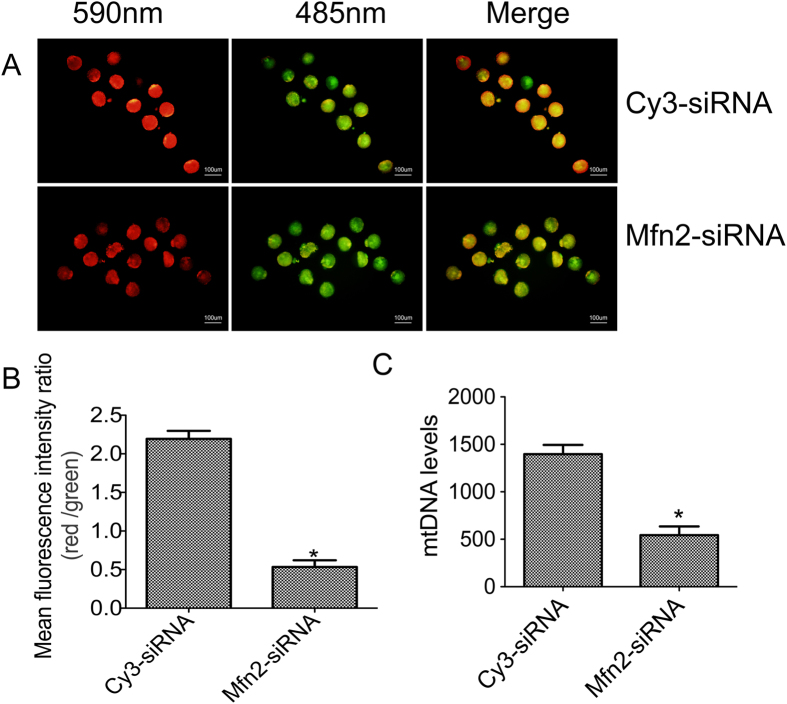
Mfn2-siRNA affects the mitochondrial function of oocytes in mice. (**A**) The most representative fluorescent images of mitochondrial membrane potential (ΔΨm) was detected by JC-1 in Cy3-siRNA and Mfn2-siRNA groups (Red, high ΔΨm; Green, low ΔΨm); (**B**) The mean intensity ratio of red and green fluorescence in Cy3-siRNA and Mfn2-siRNA groups; (**C)** Relative quantity of mtDNA in the two groups. *P < 0.05 vs. Cy3-siRNA groups.

To further explore the mechanism of Mfn2 on the development of oocytes, we measured mitochondrial membrane potential by JC-1. The results indicated that the fluorescence intensity was apparently weaker in the Mfn2-siRNA group than in the Cy3-siRNA (Fig. 3A,B) (P < 0.01). In addition, we measured the mtDNA levels in oocytes in the Mfn2-siRNA group and Cy3-siRNA group by real-time PCR. The expression of mtDNA in the Mfn2-siRNA group was significantly lower than that in the Cy3-siRNA group (Fig. 3C) (P < 0.05).

### Down regulation of *Mfn2* caused abnormal distribution of mitochondria in mouse Oocytes

**Figure 4:**
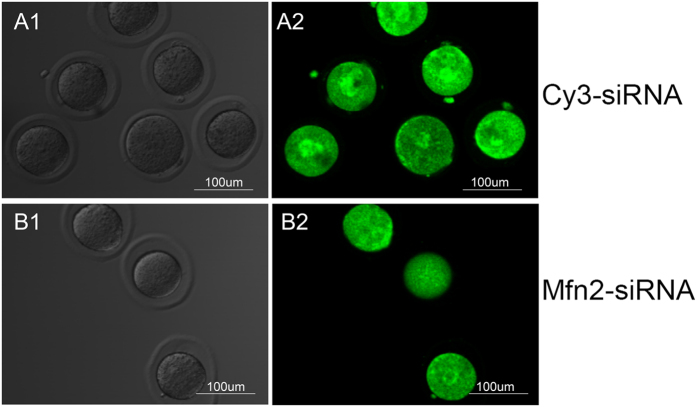
Knockdown of Mfn2 causes the morphology change and redistribution of Mitochondria. (**A1**,**B1**) The GV stage oocytes were cultured in a maturation medium and transfected with siRNA (50 nM) for 16 h, followed by immunostaining with Mito-Tracker-Green (green) in Cy3-siRNA and Mfn2-siRNA groups; (**A2**,**B2**) The most representative images for the mitochondrial redistribution of oocytes stained with Mito-Tracker-Green in two groups. In the Cy3-siRNA group, mitochondria were gathered flakily around the nucleus (69%) and scattered in the cytoplasm (31%), however, in the Mfn2-siRNA group, the mitochondria clusters around the nucleus decreased (38.9%, *P* < 0.05), while scattered distribution in the cytoplasm increased (61.1%, *P* < 0.05). Date were means ± SEM of three separate experiments.

To determine the effect of Mfn2 on mitochondria in oocyte, we observed the mitochondrial distribution patterns during the maturation of oocytes. The MII stage oocytes matured *in vitro* were stained with Mito-Tracker Green and a redistribution of mitochondria were examined by fluorescent measurement. We found, in the Cy3-siRNA group, mitochondria were gathered flakily around the nucleus (69%) and scattered in the cytoplasm (31%), (Fig. 4A1,A2). However, in the Mfn2-siRNA group, the mitochondria distribution was differently apparently, mitochondria clustering around the nucleus were decreased (38.9%, *P* < 0.05), while scattered distribution in the cytoplasm were increased (61.1%, *P* < 0.05) (Fig. 4B1) (Table 3).

### Knockdown of *Mfn2* alternated oocytes meiosis in mouse oocytes

**Figure 5:**
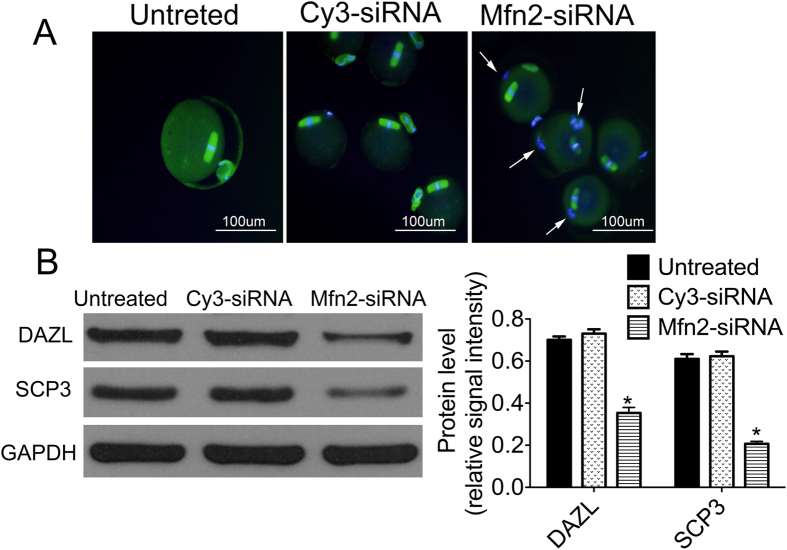
The change of oocytes meiosis and relative gene expressions in untreated, Cy3-siRNA and Mfn2-siRNA groups. (**A**) Representative images of spindle and chromosome alignment immunostained with anti-α-tubulin monoclone antibody (green) and Hochest 33258 (blue) by using confocal microscope in the three groups (Arrows, abnormal chromosome alignment and spindle with few microtubules); (**B**) The relative expression levels of meiosis-related genes *DAZL* and *SCP3* in the three groups. *P < 0.05 vs. Untreated and Cy3-siRNA groups.

In order to explore the mechanism of Mfn2-influenced oocyte development, we investigated spindle morphology and the expressions of proteins related to meiosis. When compared to the Cy3-siRNA group and untreated group, we observed a significant change in spindle morphology and aberrant chromosome separation, such as spindle with few microtubules, in the Mfn2-siRNA group by immunofluorescence. In addition, the microtubules were arranged in disorder and there were no separated homologous chromosomes found in spite of the first polar body eduction (Fig. 5A).

We measured the protein levels of DAZL and SCP3 in MII-stage oocytes, which were transfected with siRNA. The protein levels in the Mfn2-siRNA group were significantly decreased when compared to the Cy3-siRNA group and the untreated group (Fig. 5B) (P < 0.05).

## Discussion

The maturation of oocytes can be regulated by factors both inside and outside the cells. It is an important stage for the growth of gametes, which can directly affect the next generation^15^. The normal development of early embryos depends on the quality of the oocytes, especially considering that all the material and energy in demand is associated with the reserve capacity of the oocytes in the process of maturation and embryo development^16^,^17^. Mfn2 is a conserved dynamin-like GTPase, which is essential for regulating mitochondrial fusion, energy metabolism and apoptosis^18^. Previous studies showed that the different expression level of Mfn2 was closely related to mitochondrial morphology and function in mice^11^,^19^. However, it still remains unclear as to whether or not Mfn2 affects the maturation and quality of oocytes. In this study, we first investigated the expression of Mfn2 in immature oocytes transfected with Mfn2-siRNA, observed the extrusion of the first polar body and implemented IVF. Results indicated that the first polar body extrusion and fertilization rate were decreased, implying that down regulation of Mfn2 can influence the development and quality of oocytes.

Mitochondria, the main dynamic organelles in the cytoplasm, play an extraordinary role in the maturation and development of oocytes with a constant process of fusion and fission^20^. Changes in the quantity and distribution of mitochondria will inevitably influence the quality of oocytes. Many studies have proved that the normal oocytes meiosis depends on normal mitochondria distribution^21^,^22^. The mitochondrial distribution likely reflects the local ATP demands during oocyte development, and may serve as a marker of cytoplasmic maturation and proper cellular mechanisms, such as transcription, translation and nuclear maturation. The polarity of mitochondria are located in direct proximity to oocyte zona pellucida in the GV stage oocyte, and then majority mitochondria redistributed into the central patterns in oocytes after *in vitro* maturation^23^. This mitochondrial redistribution state ensures enough energy produced for the requirements of the spindle gather and PB1 extrusion. Our results show, in Mfn2 deficient oocytes of MII stage, the mitochondrial distribution was abnormal, mitochondria clustering around the nucleus were decreased and scattered distribution in the cytoplasm were increased. This abnormal mitochondria distribution leads to no enough energy for spindle formation and PB1 extrusion and then resulted in meiosis obstacle.

Mitochondrial membrane potential, which is closely related to ATP generation, is the necessary prerequisite to maintain the metabolic regulation and election transport chain during the maturation of oocytes. Therefore, the low production level of mitochondrial membrane potential will inevitably lead to a reduced maturation rate of oocytes^24^,^25^,^26^. In this study, we measured the mitochondrial membrane potential by JC-1 and measured the level of mtDNA by using real time-PCR. The results show that in the Mfn2-siRNA group, both the mitochondrial membrane potential and mtDNA level had decreased significantly. Our results suggest that low *Mfn2* expression leads to a significant effect on the quality of oocytes by regulating the morphology, quantity and eventually resulting in mitochondrial dysfunction.

The gene *DAZL* plays a vital role in the differentiation and maturation of the gamete throughout the entire process of oocytes meiosis^27^. Previous studies showed that *DAZL*-deficient mice were infertile and that the knockdown *DAZL* could cause the inhibition of maturation in GV stage oocytes, and even resulted in the fertilization failure of mature oocytes^28^. DAZL protein exists in the nucleus and cytoplasm of each developmental stage of germ cells^29^. We measured the expression level of DAZL protein by western blotting, showing that DAZL expression in the Mfn2-siRNA group was significantly lower than that in the Cy3-siRNA and untreated groups. Furthermore, our results also showed the expression of synaptonemal complex protein 3(SCP3) was significantly decreased in the Mfn2-siRNA group, indicating that SCP3 is crucial for maintaining the structure of chromosomes during the first meiotic division (meiosis I)^30^. More importantly, a significant change in spindle morphology and no separated homologous chromosomes in MII-stage oocytes were found to correlate with down regulation of Mfn2 in GV stage oocytes. These results illustrate that Mfn2 depletion can block the maturation process of oocytes resulting in abnormality.

This study provides a new discovery—low expression of *Mfn2* influences the development of immature oocytes *in vitro*, suggesting that *Mfn2* might be associated with oocyte quality by regulating mitochondrial function and oocyte meiosis. Further investigation is needed to focus on addressing the precise mechanism of Mfn2 regulation meiosis signaling.

## Materials and Methods

All *in vitro* operations were conducted under constant temperature control (37 ± 1 °C).

### Animals

This study was approved by the Animal Research Center of the Huazhong University of Science and Technology. All animal manipulations were performed according to the guidelines of the Animal Care and Use Committee. 4-week-old ICR female mice and 10–12-week-old ICR male mice were purchased from the Center for Disease Control and Prevention, Hubei province, and raised in an isolated room under controlled lighting (12 h light: 12 h dark), temperature (25 ± 3 °C) and humidity (50 ± 5%) with free food and water.

### Oocytes collection and culture

The germinal vesicle (GV) oocytes, surrounded by more than three layers of corona cumulus, were collected by puncturing the ovarian follicles of female mice with 10 IU pregnant mare serum gonadotrophin (PMSG, The Bohn Pharmaceutical Co, Ltd., China) via intraperitoneal injection after 46–48 h. Freshly isolated Cumulus-Oocytes Complexes (COCs) were cultured in 5% CO_2_ equilibrated α-MEM (Gibco, USA) and 0.2 IU/ml FSH (Livzon Group, China), 10% FBS (Gibco, USA) in an incubator at 5% CO_2_ and 37 °C. The first polar body (PB1) would extrude after IVM 16 h.

### Semen preparation, IVF and embryo culture

Non-activated sperm were collected from the cauda epididymidis of fertile male mice, and then transferred to 5% CO_2_ equilibrated IVF-30 (Vitrolife, Sweden) and incubated 1 h to activate the sperm. The semen samples with a concentration of 10^5^–10^6^ sperm cells/ml were added to 40 μl/droplet of IVF-30 containing MII oocytes matured *in vitro*. The gametes were kept together for 4–6 h at 5% CO_2_ and 37 °C in the incubator. After insemination, oocytes were removed into new embryo culture medium droplets (10 embryos/40 μl). Two-cell embryos were observed after IVF 24 h.

### siRNA mediated *Mfn2* knockdown

The chemically modified Mfn2 (5′-chol + 2′ OMe) and control (5′-chol + 2′ OMe + Cy3) siRNA were synthesized by RUIBO biotechnology (Guangzhou, China). The protocol of inhibiting the expression of Mfn2 has been described previously^19^. Briefly, the GV stage oocytes were cultured in a maturation medium and transfected with siRNA (50 nM) for 16 h. PCR and Western Blotting have been adopted to identify the potency of siRNA.

### Quantitative real time PCR and Western Blotting analysis

Total RNA was extracted by using Trizol ((Invitrogen) from oocytes samples. Reverse transcription was conducted by using the first strand cDNA synthesis kit (Thermo scientific, USA). Amplification cycles of PCR were subsequently applied using the LightCycler^®^ 96 SW 1.1 real-time PCR detection system (Roche, Switzerland). The SYBR Green II (Gene Copoeia, Maryland, USA) was detected simultaneously and β-actin was used as an internal control.

Each of western blotting and specific conditions were taken from reference to the NuPAGE^®^ Technical Guide (www.invitrogen.com/manuals). NuPAGE^®^ LDS sample buffer was added to prepared samples (70 oocytes per group) directly. ECL detected the resolved proteins and digitized autoradiographs were assembled using Photoshop.

### Detection of spindle

After IVM, denuded oocytes conducted by hyaluronidase were fixed in Microtubules fixed liquid (PIPES 0.2 mol/L, EGTA 0.1 mol/L, Formaldehyde 2%, Triton X-100 0.1%, MgCl_2_ 0.05 mol/L, ultrapure water as solute) for 30 min and blocked in confining liquid (BSA 3 mol/L, Glycine 1 mol/L, Normal Goat Serum 2%, Triton X-100, 0.01%, supplemented PBS) for 2 h and incubated at 37 °C with 1:100 anti-α-tubulin monoclone antibody for 1 h. After three washes in confining liquid, the oocytes were incubated with 1:100 Goat-anti-Mouse-IgG-FITC at 37 °C for 1 h and stained with Hochest 33258 for 5 min, and then mounted on glass slides and detected with confocal microscope (IX71, Olympus, Japan).

### Mitochondrial membrane potential and distribution

The changes of the oocytes mitochondrial membrane potential (ΔΨm) were monitored by incubating in culture medium at 37 °C for 20 min with JC-1 (Beyotime, China). After fully washing, oocytes images were acquired using scanning microscope in selective channel for green and red (590 nm and 485 nm). The distribution of mitochondria in oocytes was labeled with Mito Tracker Green (MTG, Beyotime, China) in culture medium at 37 °C for 30 min, and measured by Laser scanning confocal microscopy.

### Quantification of mtDNA relative to nuclear DNA (Mt/N)

MII stage oocytes from each group were collected to prepare total DNA using the DNeasy Blood & Tissue Kit (Qiagen, Germany). The mtDNA content was assessed by quantification of *CoxII* relative to the nuclear gene *β-actin*. The CoxII primer sequences were as follows: forward 5′-GAGCAGTCCCCTCCCTAGGA-3′ and reverse 5′-GTCG GTTTGATGTTACTGTTGCTT-3′. Nuclear and mitochondrial DNA contents were detected by real-time PCR and PCR quantification was performed in triplicate, and the amplified transcripts were quantified using the comparative Ct method. Briefly, the Ct values were calculated according to the following equation: ΔCt = Ct_CoxII_ – Ct_β-actin_, where ΔCt is the difference in the Ct values between CoxII and β-actin. The relative quantity of DNA expression in the Mfn2-siRNA groups was compared with the Cy3-siRNA groups. Xn is calculated by the formula Xn = 2^−ΔCt^.

### Statistical analysis

Each experiment was repeated more than three times with consistent results and the data presented as means ± SEM were analyzed with SPSS.18.0. Significance of differences were set at *P* <  0.05.

## Additional Information

**How to cite this article**: Liu, Q. *et al.* Mitofusin 2 regulates the oocytes development and quality by modulating meiosis and mitochondrial function. *Sci. Rep.* **6**, 30561; doi: 10.1038/srep30561 (2016).

## Figures and Tables

**Table 1:** The first body extrusion in three groups.

Groups	Total oocytes (n)	MII stage (n)	Mature percentage (%)
Untreated group	214	174	81.3
Cy3-siRNA group	193	152	78.8
Mfn2-siRNA group	201	110	54.7*

*P < 0.05 vs. Cy3-siRNA group and untreated group.

**Table 2:** The fertilization and cleavage rate.

Groups	Total oocytes (n)	2-cell stage (n)	Fertilized percentage (%)
Untreated group	129	100	77.5
Cy3-siRNA group	121	92	76.0
Mfn2-siRNA group	118	72	61.0*

*P < 0.05 vs. Cy3-siRNA group and untreated group.

**Table 3:** Morphology and distribution of mitochondria in two groups.

Groups	Total oocytes (n)	Flakily around nucleus (%)	Scattered in cytoplasm (%)
Cy3-siRNA group	88	69	31
Mfn2-siRNA group	95	38.9*	61.1*

*P < 0.05 vs. Cy3-siRNA group.
